# Comparing Low-Dose Carvedilol Continuous Manufacturing by Solid and Liquid Feeding in Self-Emulsifying Delivery Systems via Hot Melt EXtrusion (SEDEX)

**DOI:** 10.3390/ph17101290

**Published:** 2024-09-28

**Authors:** Ožbej Zupančič, Josip Matić, Aygün Doğan, Alessio Gaggero, Johannes Khinast, Amrit Paudel

**Affiliations:** 1 Research Center Pharmaceutical Engineering GmbH (RCPE), Inffeldgasse 13, 8010 Graz, Austria; josip.matic@rcpe.at (J.M.); ayguen.dogan@rcpe.at (A.D.); alessio.gaggero@rcpe.at (A.G.); khinast@tugraz.at (J.K.); 2 Institute of Process and Particle Engineering, Graz University of Technology, Inffeldgasse 13/3, 8010 Graz, Austria

**Keywords:** solid self-emulsifying drug delivery systems (SEDDSs), liquid feeding hot melt extrusion, solid-state characterization

## Abstract

**Background/Objectives:** This study compared two pilot scale continuous manufacturing methods of solid self-emulsifying drug delivery systems (SEDDSs) via hot melt extrusion (HME). **Methods**: A model poorly water-soluble drug carvedilol in low dose (0.5–1.0% *w*/*w*) was processed in HME either in a conventional powder form or pre-dissolved in the liquid SEDDS. **Results**: HME yielded a processable final product with up to 20% *w*/*w* SEDDS. Addition of carvedilol powder resulted in a non-homogeneous drug distribution in the extrudates, whereas a homogeneous drug distribution was observed in pre-dissolved carvedilol. SEDDSs were shown to have a plasticizing effect, reducing the HME process torque up to 50%. Compatibility between excipients and carvedilol in the studied ratios after HME was confirmed via DSC and WAXS, demonstrating their amorphous form. Solid SEDDSs with Kollidon^®^ VA64 self-emulsified in aqueous medium within 15 min with mean droplet sizes 150–200 nm and were independent of the medium temperature, whereas reconstitution of Soluplus^®^ took over 60 min and mean droplet size increased 2-fold from 70 nm to 150 nm after temperature increased from 25 °C to 37 °C, indicating emulsion phase inversion at cloud point. **Conclusions:** In conclusion, using Kollidon^®^ VA64 and pre-dissolved carvedilol in SEDDS has shown to yield a stabile HME process with a homogenous carvedilol content in the extrudate.

## 1. Introduction

Solubilization of poorly water-soluble drugs is an ongoing challenge in oral drug delivery [1]. Considering that advances in drug discovery lead to increasingly more lipophilic drugs [2] with improved target specificity and potency up to even pmol/L [3], novel formulations and process approaches to improve oral bioavailability are in high demand. Enabling formulations like lipid-based delivery systems (LBDSs) are one of the most extensively studied ways to overcome oral administration barriers [4]. Specifically, self-emulsifying delivery systems (SEDDSs), a subgroup of LBDSs and typically formulated as mixtures of lipids, surfactants and co-solvents, were extensively evaluated for this purpose [5]. SEDDSs are traditionally liquid lipid pre-concentrates that spontaneously emulsify in the presence of aqueous gastrointestinal fluids, effectively solubilizing the incorporated drug in the emulsion’s oily core via rapid and homogeneous dispersion in the medium [6].

Recently, an interesting new research field of solidifying liquid SEDDSs via hot melt extrusion (HME) has emerged [4]. HME is a continuous and solvent-free process, where powders and/or liquids are introduced into the extruder barrel and are heated, melted and fused into one continuous phase along the co-rotating double screws. A comprehensive review by Zupančič et al. [4] describes SEDDS solidification via HME considering process flexibility (screw configuration, feeding regime, feeding location, die shape and diameter, process parameters, etc.), real-time quality and process control, process simulation [7] and versatile downstream possibilities and their benefits as well as shortcomings and risks of preparing a hot melt extruded SEDDS (HME-SEDDS).

Various experimental setups have been proposed in preparing HME-SEDDS [8,9,10,11]. Some opposing approaches include co-extruding SEDDSs and excipients vs. feeding them separately, solid vs. liquid feeding, liquid vs. solid lipids and lab vs. pilot scales. In a study by Silva et al. [8], solidification of SEDDS containing carvedilol (CAV) was performed via lab-scale co-extrusion by pre-adsorbing up to 20% *w*/*w* SEDDS on the solid matrix composed of microcrystalline cellulose (MCC), colloidal silicon dioxide, talc and a mixture of hydroxy propyl methylcellulose-acetate/succinate (HPMCAS) and hydroxy propyl cellulose (HPC). The mixture was processed in a vertical twin-screw hot-melt extruder, and the resulting extrudates were characterized in vitro. A similar co-extruding lab-scale study, but rather with a pure polymer matrix, was performed by Schmied et al. [9]. Three different liquid SEDDSs in 20% *w*/*w* and 30% *w*/*w* containing model drugs celecoxib, efavirenz or fenofibrate were co-extruded using Soluplus^®^ (SOL), Kollidon^®^ VA 64 (VA64), HPMC and Eudragit^®^ E with different molecular weights in the conical screw setup to yield trinary amorphous extrudates composed of the drug, the SEDDS and the polymers. Improved solubilization of the drugs, faster dissolution rate and adequate HME-SEDDS physical stability were demonstrated. Likewise, Raman Kallakunta et al. [10] performed a lab-scale single-step continuous extrusion on an 11 mm co-rotating twin-screw extruder with solid feeding of a premix of one part of mesoporous silicate Neusilin^®^ US2 matrix and two parts of solid lipids and total 4% *w*/*w* of fenofibrate. The prepared HME-SEDDS had adequate emulsification properties and physical stability, was in the amorphous state and had an improved fenofibrate solubilization in vitro. 

Expanding beyond lab-scale co-extrusion trials, a continuous pilot-scale HME in a ZSK18 co-rotating twin-screw extruder was implemented via split feeding of placebo liquid SEDDS and a polymer powder. Typical HME polymers, i.e., SOL and VA 64, were processed with 10–30% *w*/*w* SEDDS to yield amorphous extrudates with no phase separation in the solid state and spontaneously formed microemulsion with droplet sizes up to 300 nm upon emulsification in deionized water [11]. 

Despite these encouraging proofs of concept, HME-SEDDS is still an uncharted territory with experimental data lacking. As mentioned above, the number of newly discovered drugs is increasing not only in terms of lipophilicity but also in terms of potency, with therapeutic doses ranging from low milligrams to micro- and even picograms. Continuous processing of powders with drug concentrations below 1% *w*/*w* is known to cause issues in pharmaceutical manufacturing, e.g., blend inhomogeneity, content uniformity deviations and low potency of the drug product due to manufacturing loss [12]. To avoid these issues in HME, low-dose drugs could be introduced in a dissolved form into the SEDDS lipid core, eliminating the additional drug-excipient preblend preparation step and improving the content uniformity of the final product. 

The aim of this study was to compare low-dose continuous manufacturing of a model drug carvedilol (CAV). The drug is a weakly basic poorly water-soluble Biopharmaceutical Classification System class II substance with pH-dependent solubility and commercially used as oral tablets in single doses between 3.125 and 25 mg [13]. These properties make CAV suitable for the development of solid SEDDSs containing a polymer matrix for enhancing CAV solubilization as well as diminishing its pH-dependent solubility by incorporating it in the SEDDS lipid core. Additionally, CAV is a well-characterized and relatively safe substance, which was used in previous studies for development of conventional SEDDSs [14], amorphous solid dispersions [15] or hybrid polymer–lipid formulations via HME [8,13] and was thus chosen as a suitable model drug for the current study.

First, CAV in final doses of 0.5–1.0% was introduced into HME via conventional preblending, with the polymer matrix and blank SEDDS introduced in the secondary HME feeding stage. Second, the blank polymer matrix wax was introduced into HME via conventional solid feeding, followed by secondary feeding of CAV-loaded SEDDS, to test a different feeding strategy and its effect on the final product. The prepared extrudates were characterized by the CAV content of uniformity, solid state and in vitro properties, such as emulsification and reconstitution time, droplet size, polydispersity index (PDI) and transmittance. 

## 2. Results and Discussion

### 2.1. CAV Solubility, Liquid SEDDS Preparation and CAV Loading in the SEDDS

The SEDDS composition and the model drug were chosen based on our previous pilot-scale study [11], data available in the literature and CAV solubility reported in single lipids. The reported CAV thermodynamic solubility in middle-chain triglycerides (caprylic/capric triglycerides) ranged between 0.73 mg/mL and 4 mg/mL [16,17], in Capmul^®^ MCM from 31 mg/g [18] to 64.26 mg/mL [17], in Kolliphor^®^ RH40 80 mg/g [18] and in Transcutol^®^ 68.42 mg/mL [17]. Considering these values and the formulation composition of 30% Kolliphor^®^ RH40, 30% Capmul^®^ MCM, 30 g Labrafac^®^ lipophile WL 1349 (medium chain triglycerides) and 10% Transcutol^®^, a simple additive calculation of partial solubility in single oils would yield an approximate CAV saturated solubility of 4.36–5.13 mg/g. 

Qualitative solubility studies with CAV were performed to rapidly screen the CAV solubility in SEDDS. The results showed that only at a CAV concentration of 10% *w*/*w* turbid SEDDS and undissolved CAV were visually observed after centrifugation. Upon gentle heating up to 60 °C, CAV completely dissolved, but precipitated from SEDDS upon cooling to room temperature. In contrast, at all CAV concentrations of 5% *w*/*w* or below, clear/transparent SEDDSs with no sediment after centrifugation were observed. This indicated that the CAV solubility in the SEDDSs was around 5% but below 10% *w*/*w*. After one month of storage of 5% *w*/*w* CAV SEDDS preconcentrate in a sealed glass container and protected from light, no CAV precipitation was observed. Hence, for further experiments and to achieve a CAV concentration of 1% *w*/*w* or lower in the final product, 5% *w*/*w* CAV loaded in the SEDDS was chosen. 

Characterization of the emulsions with 0.4% *w*/*v* and 2.0% *w*/*v* of blank and CAV-loaded SEDDS, as shown in Table 1, was performed to evaluate if CAV had any substantial effect on the SEDDS performance. Incorporating CAV into the SEDDS led to a significant (*p* < 0.05) increase in the mean droplet size and PDI but had no significant impact (*p* > 0.05) on the emulsification time. It was reported in the literature that increased drug loading can lead to prolonged emulsification times, an increase in the SEDDS viscosity and an overall negative effect on the droplet size and PDI. All of this could significantly affect the SEDDS in vitro performance [6]. In our study, however, both the blank and the CAV-loaded SEDDSs could be classified as Grade A, which is defined as rapidly forming (within 1 min) emulsions with a clear or bluish appearance [19]. 

### 2.2. Hot Melt Extrusion (HME)

To omit additional technical and processing difficulties during HME, the process was performed within the processing window defined in the previous study [11], in which SEDDS loadings of up to 30% *w*/*w* were achieved under the processing condition described above. However, for CAV trials, SEDDS loadings up to 20% *w*/*w* were chosen since 30% *w*/*w* yielded non-uniform extrudates with an oily consistency and a strong tendency to coalesce. 

Moreover, SEDDS loadings higher than 30% *w*/*w* yielded a lower viscosity melt and were reported to leak from the degassing opening in the eighth HME segment and failed to solidify into a processable solid strain after cooling [11]. For these reasons, the degassing was omitted within this study. Additionally, most lab-scale studies listed above considered 20% *w*/*w* SEDDS loadings in the final formulation optimal [8,9]. Moreover, a limit of up to 20% *w*/*w* lipids in the final composition was observed when using solid lipid Acconon^®^ C-50 for preparing amorphous solid dispersions (ASDs) via HME [13]. 

**Figure 1:**
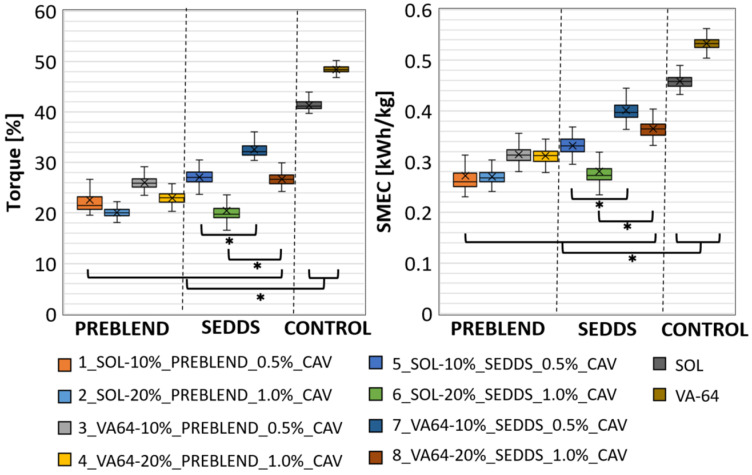
Torque (**left**) and SMEC (**right**) variability in the various BEER sample extrusions. * *p* < 0.05.

The extrudate sample collection was performed after a steady state was achieved, as signified by a constant torque value recorded during processing (Figure 1). All process settings and tested formulation variants resulted in mean torque levels between 20% and 32% of the maximal available torque. This corresponds to values of 14 Nm to 23 Nm. More important were the narrow standard deviations, which indicate low variability of the torque and, consequently, a steady-state process during the sample collection. According to the torque values, the specific mechanical energy consumption (SMEC) was 0.27 kWh/kg–0.40 kWh/kg on average, i.e., within the normal range in pharmaceutical HME processing. In addition, a plasticizing effect of SEDDS was demonstrated. The recorded process data showed that the screw torque was significantly (*p* < 0.05) reduced by increasing the SEDDS feeding rate. Considering that SEDDS and typical plasticizers used in HME [4,20] (e.g., fatty acid esters, citrate esters and vitamin E derivatives) are of comparable lipophilic nature, SEDDS is expected to demonstrate a certain plasticizing ability. Indeed, the plasticizing ability was polymer-dependent with a general trend of SOL samples showing lower process torques compared with VA-64 counterparts with the same *w*/*w*% SEDDS amount. For instance, a significant difference (*p* < 0.05) in torque was observed between 5_SOL-10%_SEDDS_0.5%_CAV and 7_VA64-10%_SEDDS_0.5%_CAV as well as 6_SOL-20%_SEDDS_1.0%_CAV and 8_VA64-20%_SEDDS_1.0%_CAV (Figure 1 left). 

A possible explanation for the general lower torques in SOL samples could likely be attributed to its lower molecular weight and subsequently lower T_g_.

**Figure 2:**
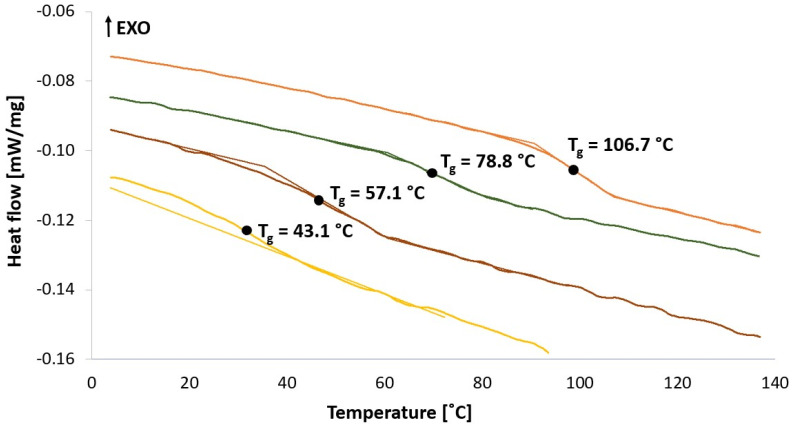
Glass transition temperature (T_g_) reduction in VA-64-HME-SEDDS via DSC (exo up). By increasing SEDDS throughput in the process and subsequently SEDDS amount in the HME-SEDDS, a trend of T_g_ reduction of very roughly 20 °C per each 10% *w*/*w* SEDDS increase was seen. No T_g_ was detected in SOL-HME-SEDDS.

Increasing SEDDS amount in HME-SEDDS led to a reduction in HME torque (Figure 1) and the glass transition temperature (Figure 2). A clear reduction in T_g_ with an increasing SEDDS content was evident in VA64, which has a T_g_ of ~106 °C. In contrast, T_g_ reduction was not detectable in SOL. The reason for indistinguishable SOL T_g_ might be that remarkably higher process temperatures of 110–140 °C are applied in HME, whereas SOL T_g_ is at about ~70 °C. These findings were supported by another study, which reported a reduction in T_g_ of SOL, VA-64, Kollidon^®^ 17 PF, Eudragit^®^ E and HPMC in HME-SEDDS formulations containing 20% *w*/*w* SEDDS [9]. Reduction in process torque and T_g_ could be beneficial if the HME process temperature should be reduced due to potential raw material degradation risk or process technical reasons. 

Moreover, the polymer matrix seemed to have a profound effect on the final SEDDS loading and the HME process itself. In another study, in which only HPMCAS was used as the polymer matrix, extrusion was not feasible. After adding hydroxypropylcellulose (HPC) to the formulation, extrusion was feasible to up to 20% *w*/*w* SEDDS. Indeed, oil binding capacity of polymers could be considered a critical formulation attribute that has a direct impact on the formulation processability [8]. If a polymer’s oil binding capacity is exceeded, semi-solid or liquid extrudates are expected, which are not optimal for downstreaming [9]. Hence, oil binding studies using binary mixtures of polymers and SEDDS are recommended in the pre-formulation phase prior to HME [4,11].

#### 2.2.1. CAV in Preblend

Although the HME process described above was by itself unaffected by the way CAV was introduced, there are certain aspects worth considering when processing low-dose formulations via HME. First, introducing CAV into the preblend required an additional blending step, which is cost- and time-intensive. Preparing the preblend was necessary since feeding the polymer and CAV separately was unfeasible as gravimetric feeders cannot precisely and consistently feed the powder at the required low feeding rates and due to poor flowability of micronized pure API powder, as is the case of CAV [12]. In addition, the high cohesiveness of CAV powder tends to result in agglomerates even in preblends, making it extremely challenging to consistently feed such a powder blend while ensuring the required content uniformity [12]. All these factors increase the risk of CAV and polymer segregation in the preblend, causing inconsistent feeding and non-uniform drug concentration in the extrudate. 

The conventional way to introduce a drug to an HME process is in the solid form. However, an obvious drawback of the preblend is the risk of drug and polymer segregation, causing inconsistent feeding and non-uniform drug concentration in the extrudate. Noteworthy, segregation can be induced by the vibrations caused by the extruder itself. Prior to preparing the preblends, the PSD of CAV, SOL and VA-64 was assessed. Table 2 shows that preblends containing 0.5–1.0% *w*/*w* CAV will likely undergo segregation due to significant differences (*p* < 0.05) in the mean particle size between CAV and the polymers. The highest segregation is expected in the preblends containing SOL due to an almost 30-fold higher D_50_ compared to CAV.

#### 2.2.2. Carvedilol in the SEDDS

Considering the shortcomings of processing CAV in the preblend, an alternative, i.e., feeding of CAV dissolved in the SEDDS during HME, was evaluated. To the best of our knowledge, liquid feeding in HME has not been widely explored to date, specially using liquid lipids as the vehicle. In one study, liquid feeding of a viscous aqueous suspension containing 1% *w*/*w* ibuprofen, 35% *w*/*w* PEG 20.000 and 65% deionized water in a premix of copovidone/povidone (80:20) was performed on the same ZSK18 twin-screw co-rotating extruder as the one used in this study. The homogeneity and relative standard deviation (RSD) of extrudates containing 0.021–0.043% *w*/*w* ibuprofen was found to be between 2 and 7%. The water introduced was removed in situ via devolatilization [12]. In this study, the secondary liquid feeding was performed using the SEDDS as the lipid vehicle in the fourth segment of HME. The rationale behind this was to add SEDDS to a completely melted polymer matrix to ensure homogenous mixing. Moreover, a shorter exposure of CAV to high process temperature may reduce the potential drug degradation during HME. Apart from that, advantages of liquid feeding of low-dose drugs include a straightforward single step and a precise process control as liquid pumps show accurate feeding rates down to µL/h even for low dosing volumes. This advantage can already be utilized in the pre-formulation drug product development stages, when only small amounts of the drug candidate are available [21]. Besides omitting the blending unit operation, introducing accurately controllable liquid pumps in the HME provides an additional monitoring parameter via PAT tools for more efficient scale-ups, process control and quality assurance [12].

Beyond the content uniformity and process benefits described above, additional features of liquid feeding in HME could be applied in the formulation strategy. Split feeding of lipids and polymers can be performed to tackle the issue of incompatibility between drugs, excipients or lipids when co-extrusion is not feasible [11]. Furthermore, reducing the residence time of heat-sensitive drugs in HME may improve the drug product’s chemical stability [4]. Finally, dissolving one or more components in SEDDS when processing drugs and excipients with massive differences in glass transition temperature (T_g_) could contribute to homogeneous mixing of the components in the extrudate. For example, incompatibilities between VA-64 and a solid lipid Acconon^®^ C-50 and a phase separation in the final extrudate were observed when both materials were processed via solid feeding in a preblend. Substantial differences between a polymer and a lipid prevented homogeneous mixing and fusion between the lipid and the polymer below processing temperatures of 100 °C [13].

With regard to oral drug delivery, introducing lipids into a formulation would improve oral bioavailability of poor water solubility of BCS class II drugs [4]. A formulation shortcoming of HME-SEDDS is that the drug concentration in the extrudate is limited to its saturated solubility in SEDDS and polymer matrix. Feeding drug particles suspended in SEDDS would be a viable option if the SEDDS viscosity would be appropriate to ensure a homogeneous particle dispersion in the lipid suspension, which would result in uniform drug content in the HME-SEDDS [12]. Alternatively, feeding a heated SEDDS solution to solubilize the suspended drug, as described above for the SEDDS loaded with 10% CAV, could be considered.

#### 2.2.3. Comparing CAV Introduced to HME via Solid and Liquid Feeding

Table 3 shows CAV recovery in the HME-SEDDS samples prepared in liquid feeding setups, where CAV was either included in the preblend or dissolved in the SEDDS.

**Figure 3:**
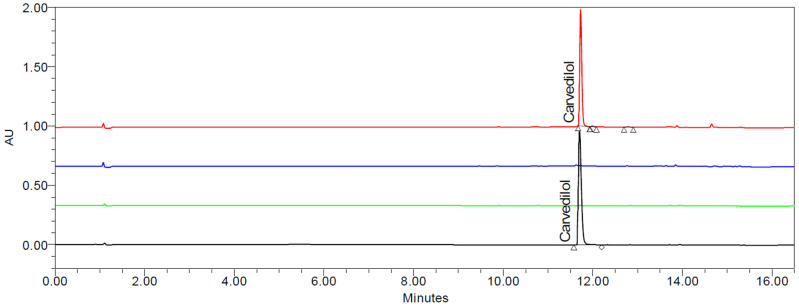
Typical chromatograms of CAV standard (black), blank mobile phase (green), excipient matrix without CAV (blue) and 5_SOL-10%_SEDDS_0.5%_CAV sample (red).

To prove the results’ reliability, specificity and accuracy data of the analytical method are provided in the method section. Blank mobile phase and matrix injections did not show any interference with the main CAV peak, and the purity was confirmed by the “Purity Flag” tool. The typical chromatograms for CAV standard, blank mobile phase, blank sample matrix and a typical HME-SEDDS sample containing CAV are presented in Figure 3.

Since the HPLC method’s accuracy was verified, the erratic results may be attributed to the preblend’s homogeneity and a significant difference in the PSD between CAV and the polymers, leading to segregation. The most contradictory result is 2_SOL-20%_PREBLEND_1.0%_CAV, where only 37.04% of CAV was recovered in the preblend. Moreover, the HME-SEDDS in the same sample showed 53.29% CAV. The erratic results in the preblends could likely be attributed to the above-described processing issues related to low-dose HME processing. Moreover, sampling low-dosed powders and sample preparation techniques like sample splitting and CAV extraction from the matrix should not be neglected. However, the recovery of CAV in samples, where CAV was dissolved in SEDDS, was more encouraging compared to the preblend samples, except for 5_SOL-10%_SEDDS_0.5%_CAV, which also has the highest STD of 10.47%. Nevertheless, based on the UPLC analytical method development data, the CAV content per single dose will be reliable, provided the manufacturing HME process, where CAV is introduced in the dissolved form via SEDDS, is developed in accordance to GMP and other chosen oral solid dosage form compendial quality requirements.

The aim of this study was not drug product development with an intrinsically complex process, but rather a proof of concept and a head-to-head comparison between two different approaches to preparing low-dose formulations via pilot-scale HME using lipid SEDDS as the vehicle in the liquid feeding setup. Noteworthy, in this specific case, the expected CAV commercial dosage form would be a type of oral solid like powder, pellets, hard gelatin capsules or tablets. The dosage form should be enteric-coated to prevent possible CAV precipitation during pH shift from an acidic into a neutral small intestinal pH environment. Considering the commercial CAV product containing 3.125, 6.25, 12.5 or 25 mg CAV per single dose, HME-SEDDS prepared within this work containing 1.0% *w*/*w* CAV could be formulated in tablets with total weight up to 500–750 mg or filled into size 0 or 00 hard gelatin capsules containing 312.5 mg HME-SEDDS and 3.125 mg CAV, respectively.

The results of the study are, in our opinion, a pioneering step in establishing HME as an alternative solidification technique for lipid-based delivery systems, in particular SEDDS. HME solidification could be used instead of spray-drying, freeze-drying or adsorption on (meso)porous adsorbent carriers [22], where liquid lipids would cause technical issues in solidification. HME offers solvent-free solidification without pre-emulsification of SEDDS in the aqueous phase, preserving the intact self-emulsification ability of the formulation. Pre-emulsification of SEDDS may cause the loss of microemulsion integrity by passing the cloud point at applied process temperatures, causing alterations of self-emulsification performance as well as potential loss of CAV solubilization capacity after oral administration.

### 2.3. HME-SEDDS Solid-State Characterization

#### 2.3.1. Organoleptic Evaluation

**Figure 4:**
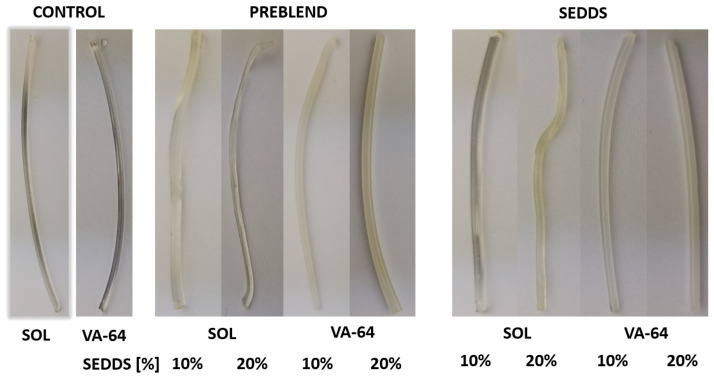
HME-SEDDS appearance after extrusion and cooling.

The extrudates were evaluated organoleptically in terms of appearance, color and any other visible properties. Figure 4 illustrates the prepared HME-SEDDS on a macroscopic level.

Neither the method of introducing CAV nor including up to 1.0% *w*/*w* CAV in the final product resulted in significant visual changes in the extrudates compared to the blank HME-SEDDS prepared previously [11]. The extrudate properties were polymer-driven: the SOL samples were generally more transparent and elastic, whereas VA-64 samples were brittle and wax-like yellowish-white. A transparent extrudate appearance via HME indicates an amorphous drug state and a homogenous distribution of the drug within the polymer matrix, also signifying the compatibility of the materials and the absence of degradation. However, since preparing HME-SEDDS (or other lipids) is still in its infancy, this empirical rule may not apply.

#### 2.3.2. Differential Scanning Calorimetry (DSC) and Wide-Angle X-ray Scattering (WAXS)

**Figure 5:**
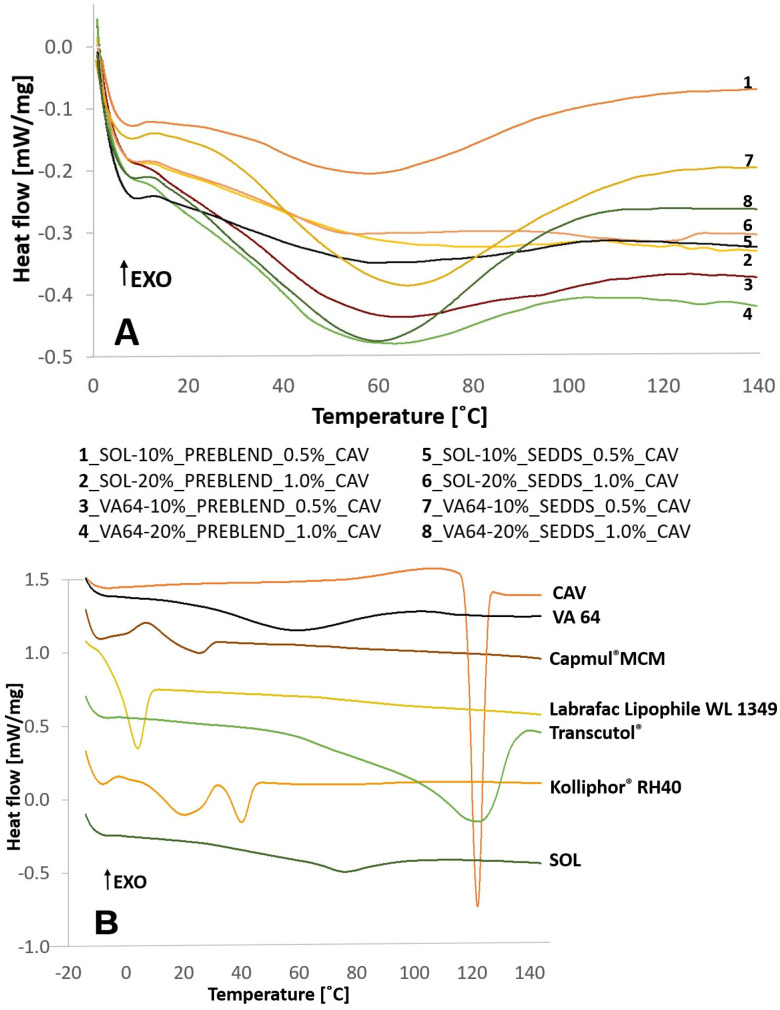
(**A**) DSC of HME-SEDDS containing CAV and (**B**) single raw materials used in the formulation.

**Figure 6:**
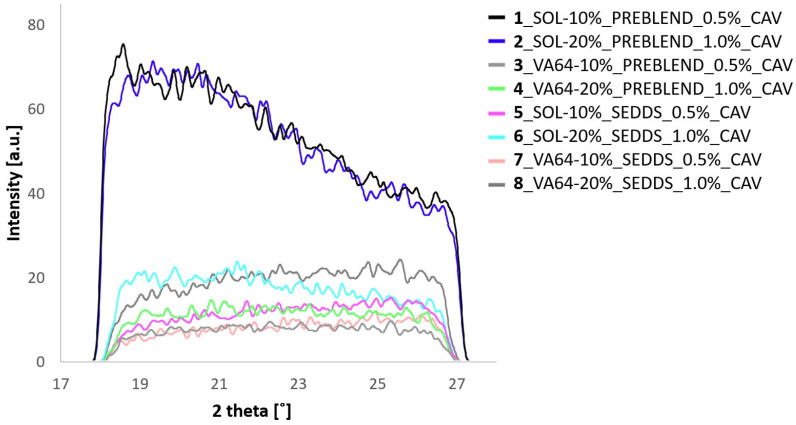
Overlay of WAXS patterns of different HME-SEDDS.

Figure 5 and Figure 6 show DSC thermograms and WAXS spectra of the prepared samples.

Figure 5 and Figure 6 indicate an amorphous state of all HME-SEDDS and no phase separation in the samples as confirmed by DSC and WAXS, regardless of the CAV introduction method, the polymers used or the SEDDS concentration in the formulation. In addition, all samples were amorphous, with no polymer, lipid or CAV crystallization observed. The amorphous state of prepared HME-SEEDS was also confirmed in the previous study that used blank HME-SEEDS with the same materials and SEDDS up to 30% *w*/*w* [11]. In view of the low dose (0.5–1.0%) of incorporated CAV, it is not surprising that CAV was successfully amorphisized in the polymer-SEDDS matrix. Additionally, according to the literature, binary amorphous solid dispersions with a pure polymer matrix or even trinary systems including polar lipids can be prepared with CAV loadings of up to 20% [10]. On this note, follow-up studies of kinetic amorphous state storage stability of high-dose CAV in the polymer matrix with added SEDDS would be of great interest. Such a study, but with a lower drug loading of up to 6.3%, was performed recently, with DSC thermograms confirming the amorphous state of extrudates containing 20% *w*/*w* SEDDS after 6 months of storage in sealed containers at 30 °C/65% RH [9]. Indeed, introducing SEDDS, lipids or surfactants to ASDs prepared via HME is being more widely considered. However, prior to HME, it is recommended to evaluate the compatibility of the drug, polymer and lipid components using such established methods as melt casting, film casting, polarized light microscopy, DSC and X-ray diffraction [4,23].

### 2.4. In Vitro Characterization

#### 2.4.1. Reconstitution Time

**Figure 7:**
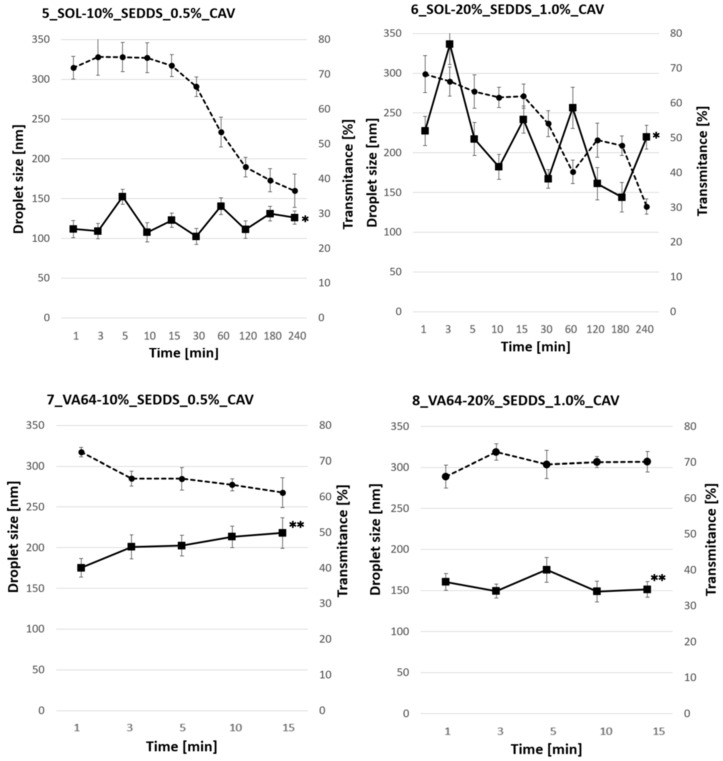
Reconstitution of 0.4% *w*/*w* HME-SEDDS in which CAV was introduced in the dissolved form. Emulsification properties were assessed at-line during reconstitution. The full and dashed lines represent the mean droplet size ± SD and the transmittance ± SD and were automatically calculated with equipment software Kalliope^®^ version 2.22.2 (Anton Paar, Graz, Austria). * *p* < 0.05 between SOL samples, ** *p* < 0.05 between VA-64 samples.

Due to the elastic nature of SOL samples, manual micronization of extrudates with a mortar (as in the case of VA64 samples) was not feasible. Rather, cryomilling was applied to the HME-SEDDS in which CAV was introduced in the dissolved SEDDS form. Figure 7 shows a reconstitution of 0.4% HME-SEDDS in deionized water at 37 °C.

It was established that the reconstitution time was polymer-driven, with the SOL samples requiring more than 240 min to completely emulsify. In contrast, VA-64 samples self-emulsified within 20 min. The impact of SEDDS amount in HME-SEDDS in the formulation on the emulsification time was negligible. The difference between 7_VA64-10%_SEDDS and 8_VA64-20%_SEDDS emulsification time was non-significant (*p* < 0.05), with average emulsification times of 10 ± 3 min and 15 ± 4 min, respectively. The variability in emulsification times could be attributed to the use of cryomilled powders, which tended to aggregate and float on the dissolution medium. In qualitative terms, interesting trends were observed during reconstitution. Generally speaking, VA-64 showed less changes in the droplet size and transmittance values. Within 15 min of emulsification time, both the droplet size and the transmittance remained in the ranges between 150 and 200 nm and 60 and 70%, respectively. In contrast, 5_SOL-10%_SEDDS_0.4% and 6_SOL-20%_SEDDS_0.4% showed significant (*p* < 0.05) variation in the droplet sizes in the measured time points, the largest being in 6_SOL-20%_SEDDS_0.4%, where shifts between 150 nm and 350 nm were recorded. In addition, a common trend of decreasing transmittance was characteristic of both SOL samples, and after 240 min, it remained constant between 30 and 40%.

Reconstitution times are not to be neglected when developing solid SEDDS for oral delivery. Obviously, solidified SEDDS formulations have longer emulsification times than their liquid SEDDS counterparts due to the additional dissolution step required. In any case, emulsification time should be rapid enough to allow homogeneous and uniform emulsification leading to a small droplet size and a narrow polydispersity index, which guarantee consistent dissolution and drug release from the formulation. In addition, given that an average intestinal transit time is about 4 h, SEDDS should be able to solubilize and release the drug within this timeframe in order not to pass the drug absorption window. Previously developed HME-SEDDS had a sub-optimal reconstitution time of up to 5 h, which is not recommended for oral delivery [8].

Indeed, a dissolution study would be the next essential step in evaluating the formulation in vivo performance after oral administration. However, performing conventional compendial USP 2 apparatus dissolution would in this case be an unsuitable approach due to low CAV dose. Dissolution experiments should be performed under biorelevant conditions, using ideally an in vitro transfer model with a pH shift from acidic into small intestinal pH (increase from 1.2 to 6.8) as well as using low dissolution medium volumes up to 200 mL simulating dosage form administration with a glass of water to achieve non-sink conditions and in vivo emulsification concentrations [24].

#### 2.4.2. Emulsification Properties of HME-SEDDS

**Figure 8:**
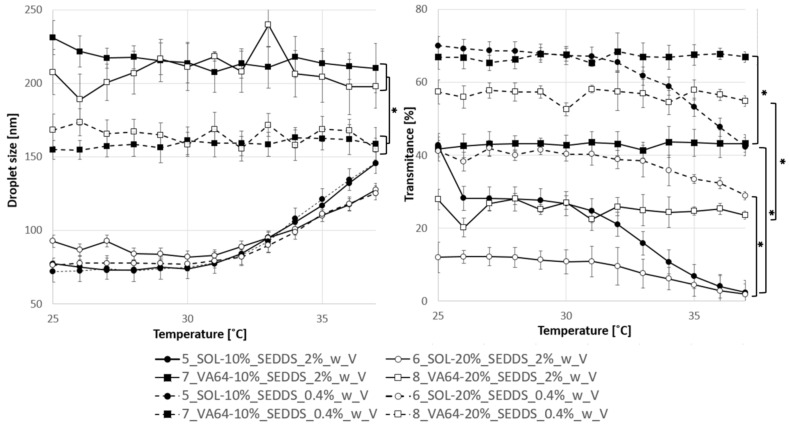
Droplet size and transmittance change in 0.4% and 2% *w*/*v* emulsions of HME-SEDDS during the temperature increase from 25 °C to 37 °C. ■ = VA-64, ● = SOL, full line = 2.0%, dashed line = 0.4%, black = 10% *w*/*w* SEDDS, white = 20% *w*/*w* SEDDS. Mean values ± SD were automatically calculated with equipment software Kalliope^®^ version 2.22.2 (Anton Paar, Graz, Austria). * *p* < 0.05.

Figure 8 shows emulsification properties of HME-SEDDS with CAV introduced in solubilized form into the SEDDS prepared via cryomilling in the final concentrations of 0.4% *w*/*v* and 2.0% *w*/*v*. Mean droplet size in SOL samples was independent of the concentration as well as SEDDS *w*/*w* % amount in the final formulation and was gradually increasing with temperature from about 75 nm to 150 nm at 37 °C. On the contrary, the emulsification properties of VA-64 samples were independent of the temperature but showed significant differences (*p* < 0.05) in mean droplet sizes between 0.4% *w*/*w* and 2.0% *w*/*w* concentrations. The diluted 0.4% *w*/*v* samples had mean droplet sizes between 150 and 170 nm within the entire measured temperature range of 25–37 °C, whereas the 2.0% *w*/*w* samples were significantly higher within the 200–230 nm range. In addition, significant differences (*p* < 0.05) in emulsion transmittance were observed. Polymer matrix, SEDDS *w*/*w*% amount and final emulsion concentration significantly affected emulsion transmittance, where VA-64-based HME-SEDDS, 0.4% *w*/*w* concentrations and 10% *w*/*w* SEDDS amount in HME-SEDDS were significantly more transparent than SOL-based HME-SEDDS, 2.0% *w*/*w* concentrations and 20% *w*/*w* SEDDS, respectively.

## 3. Materials and Methods

### 3.1. Materials

Carvedilol (99.67%) was purchased from Dacon Natural Products (Beijing, China). Kollidon^®^ VA-64 (copovidone), Soluplus^®^ (Poly(vinyl caprolactam-covinylacetate-ethylene glycol) graft polymer) and Kolliphor^®^ RH40 (PEG-40 Hydrogenated Castor Oil) were received from BASF SE (Ludwigshafen am Rhein, Germany). Capmul^®^ MCM EP (Glycerol Monocaprylocaprate Type I EP) was provided by Abitec Corp (Janesville, WI, USA). Transcutol^®^ (diethylene glycol monoethyl ether) and Labrafac^®^ lipophile WL 1349 (medium chain triglycerides) were received from Gattefossé (Saint-Priest, France). The same raw materials and formulation compositions as listed in Table 4 were used as previously [11].

### 3.2. Carvedilol Solubility, Liquid SEDDS Preparation and Carvedilol Loading in SEDDS

Liquid blank SEDDS were prepared as described previously [11]. First, semi-solid Kolliphor^®^ RH40 was melted in a water bath (50 °C) for 30 min. Next, 30 g of Kolliphor^®^ RH40, 30 g of Capmul^®^ MCM, 30 g of Labrafac^®^ lipophile WL 1349 and 10 g of Trancutol^®^ were added to a 250 mL glass beaker and stirred overnight at 200 rpm using a magnetic stirrer Heildorph MR HEI Standard (Schwabach, Germany).

CAV solubility in the SEDDS was estimated qualitatively as follows: 1.0% *w*/*w*, 2.5% *w*/*w*, 5.0% *w*/*w* and 10.0% *w*/*w* CAV were dispersed in the liquid SEDDS. The samples were left to equilibrate at room temperature in sealed Eppendorf tubes and were protected from light. After 48 h, the samples were centrifuged in a Hettich Universal 320 R (Tuttlingen, Germany) at 10,000 rpm for 10 min to separate the undissolved CAV from the SEDDS. No sediment indicated complete dissolution of CAV. The highest CAV loading with no sediment observed after centrifugation was used for further studies.

### 3.3. Preblend Preparation

Preblends of CAV with SOL or VA 64 in two final CAV concentrations of 0.5% *w*/*w* and 1.0% *w*/*w* were prepared as follows: the polymer and CAV were weighted into a plastic container through a 800 µm sieve to de-lump the CAV clusters. Next, the sieved mixture was blended for 20 min at 60 Hz in a T2F Turbular blender (Willy A. Bachofen Maschinenfabrik, Nidderau, Germany).

### 3.4. Particle Size Distribution (PSD)

Particle size distribution (PSD) of CAV, SOL, VA-64 and other samples was measured via a dynamic picture analysis (Sympatec QICPIC, Clausthal-Zellerfeld, Germany) using a dry disperser (RODOS) with a 10 mm injector. The distribution width (span) was calculated in Equation (1) as follows:

(1) $$span=\frac{d90-d10}{d50}$$

### 3.5. Hot Melt Extrusion

**Figure 9:**
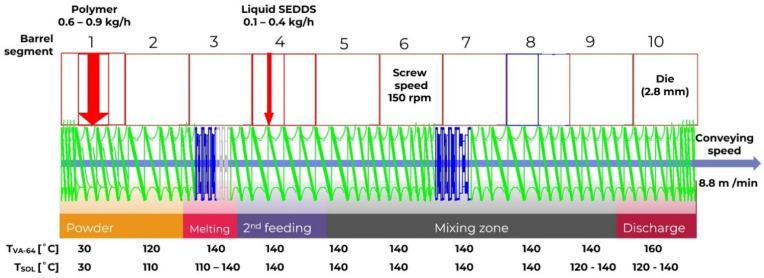
The screw configuration used in the SEDDS trials with a ZSK18 co-rotating twin-screw extruder from Coperion.

The HME setup described in detail in [11] was used. In brief, a ZSK18 co-rotating twin-screw extruder (Coperion GmbH, Stuttgart, Germany) with one feeder (in the case of preblend) and in a split feeding setup (in the case of liquid feeding of SEDDS with or without CAV) was used. HME-SEDDS samples were prepared as follows: The pure polymer powder or its preblend with CAV was introduced into the process via a K-Tron KT20 feeder (Coperion GmbH, Germany) into the first extruder segment and the liquid SEDDS with a calibrated peristaltic pump Ismatec MV-CA8 (Zürich, Switzerland) into the fourth segment. The HME process began by extruding the pure powders (pure polymers or preblends with CAV) with total throughput 1 kg/h. After steady state was reached (10–15 min), the extrudates were sampled, and the feeding ratios between solids and liquid were adjusted to yield HME-SEDDS with increasing amount of SEDDS, namely 10, 20, 30 and *w*/*w*. The feeding rate of solids was accordingly adjusted to keep the total throughput of 1 kg/h. After each SEDDS throughput increase and achieved steady state, HME-SEDDSs were sampled. Immediately after HME, a conveyor belt and an adapted cooling air tunnel from Dorner GmbH (Thalmässing, Germany) were employed to facilitate the extrudate cooling and minimize its sticking on the conveyer belt. The process data were collected in real time using XamControl software version 1.2 (Evon GmbH, St. Ruprecht an der Raab, Austria). Essential HME process settings of individual processing barrel segments are shown in Figure 9.

The powder intake zone was located at the beginning of the screw configuration with the aim to (1) provide a high free volume to increase the amount of powder intake from the first powder feeder; and (2) densify the powder for the upcoming melting zone. The screw configuration of so-called “Schubkanten” (special conveying elements) and standard conveying elements with pitches from 36 mm to 16 mm before the melting zone were used. The melting zone was designed based on preliminal process simulations to completely melt the solids. For this purpose, the melting zone contained a set of 45° kneading elements (KB 45/5/8) and a single 90° kneading element (KB 90/5/16). This combination of kneading elements should result in a high fill level, and thus a sufficient residence time in this zone, due to the 90° kneading element and the breakup of any potential agglomerates by combining the kneading elements with various kneading block thicknesses.

In the case of liquid feeding, the peristaltic pump fed into the conveying section with conveying elements of a 36 mm pitch. The large pitch allowed higher intake flow rates from the secondary feeding. The mixing section was designed as a combination of two 45° kneading elements, KB 45/5/8 and KB 45/5/16. This setup should result in a sufficient mixing action without extensive shear input. Following the mixing zone, the screw configuration was set up as the extrudate discharge zone. The discharge zone contained low-pitch conveying elements of 16 mm and 8 mm that ensured an efficient pressure build-up before the die section and a continuous extrudate discharge.

The formulations prepared by CAV addition in the preblend (Samples 1–4) or dissolving CAV in SEDDS (Samples 5–8) are shown in Table 5.

### 3.6. Cryomilling

Due to the elastic nature of some samples, the extrudates were cryomilled using Cryomill Retch (Haan, Germany) to evaluate the in vitro performance of micronized powders and potential downstreaming possibilities. Briefly, 7 g of the crushed extrudate was placed in the cryomill with the stainless-steel ball. An inbuilt default program P2 was applied with one cooling cycle of 80 s, an amplitude of 25/s and pre-cooling in a 5/min automatic mode. The micronization process was performed at −80 °C using liquid nitrogen. The milled samples were placed into sealed plastic containers protected from light until further use.

### 3.7. Solid-State Characterization

#### 3.7.1. Differential Scanning Calorimetry (DSC)

The thermal analysis of raw materials and HME-SEDDS was performed in a Netzsch DSC 204F1 Phoenix (Tirschenreuth, Germany) with an auto-sampler. Briefly, 5–10 mg of the samples were enclosed in pierced 40 µL aluminum pans. The extruded samples were analyzed using the modulated mDSC method, i.e., heated from 0 °C to 150 °C at a rate of 5 °C/min, for a period of 40 s and at an amplitude of 0.531. Raw materials were analyzed via a similar method, only the temperature was varied from −20 °C to 150 °C in order to capture the melting peak of liquid lipids. In addition, to remove the water peak from the DSC thermograms, the following gradual heating–cooling–heating cycle DSC method was used: heating from 0 °C to 150 °C at 10 °C/min, holding on 150 °C for 2 min; cooling from 150 °C to 0 °C at 10 °C/min, holding at 0 °C for 5 min; and finally heating from 0 °C to 150 °C at 5 °C/min. A period of 40 s and an amplitude of 0.531 were kept constant throughout the measurements.

#### 3.7.2. Wide-Angle X-ray Scattering (WAXS)

WAXS measurements of the HME-SEDDS were performed using previously described methods [11]. Briefly, the samples were measured at room temperature in a Hecus S3-MICRO system (Bruker Corporation, Billerica, MA, USA) equipped with two linear position sensitive detectors (2Hecus PSD-50, 54 μm/channel). The samples were placed inside X-ray capillaries of 2 mm in diameter and rotated during exposure at ∼0.2 Hz in a temperature-controlled cuvette (TCCS and SpinCap, Hecus X-Ray Systems GmbH, Graz, Austria). Measurements of the capillaries filled with the samples were performed with an exposure time of 1200 s. The background of the empty capillary was subtracted from the actual sample measurement, and the intensities were normalized via the peak area of the primary beam, i.e., the scattering mass measured using a Tungsten filter.

### 3.8. In Vitro Characterization

#### 3.8.1. Reconstitution Time

Reconstitution time of HME-SEDDS was assessed as it commonly is for solid SEDDS, but with minor modifications [27]. First, 1.0 g of micronized HME-SEDDS powder was placed into 250 mL of deionized water at 37 °C under constant stirring at 550 rpm using a heating plate Heildorph MR HEI Standard (Schwabach, Germany). At defined time points, 1 mL of medium was withdrawn and centrifuged in a mini centrifuge Combi spin PCV-2400 (Royston, UK) for 5 s at 6000 rpm to remove the not-yet-emulsified dispersed solids. The supernatant was analyzed at-line in terms of droplet size, polydispersity index (PDI) and transmittance in a Litesizer 500 (Anton Paar, Graz, Austria) as described above. The sediment was carefully diluted with 1 mL of deionized water and returned to the original 250 mL medium.

#### 3.8.2. Droplet Size, Polydispersity Index (PDI) and Transmittance

Samples for the droplet size and PDI determination were prepared as follows: 100 mg (2% *w*/*v*) or 20 mg (0.4% *w*/*v*) of HME–SEDDSs were emulsified in deionized water via gentle agitation until uniform microemulsion was observed. Next, 0.5–1.0 mL of the prepared microemulsion were analyzed in the Litesizer 500 in the automatic mode and with a calibration time of 60 s using the backscatter measurement angle.

The droplet size and temperature relationship were measured in the Litesizer 500 as follows: 2.0% or 0.4% *w*/*v* HME-SEDDS were gradually heated in 1 °C steps, starting at 25 °C and until the final temperature of 37 °C was reached. During each stage, the droplet size, the PDI and the transmittance were determined. The measurements were processed and statistically analyzed for mean values and standard deviations automatically by using the Kalliope^®^ software version 2.22.2 (Anton Paar, Graz, Austria).

### 3.9. UPLC Analysis

The CAV analysis was performed in a UPLC Acquity H-Class system equipped with a Photo-Diode Array detector, with an Acquity HSS T3 1.8 µm 2.1 × 100 mm column from Waters Corp. (Milford, MA, USA) and at a flow rate of 0.3 mL/min, an injection volume of 1 µL, a column temperature of 30 °C, a detection wavelength of 240 nm. The mobile phase was 5 mM of ammonium acetate pH 4.5 (mobile phase A) and methanol (mobile phase B) with the following gradient: 0 min: 10% B; 3 min 10% B, 13.5 min 90% B; 14.5 min: 10% B; and 16.5 min 10% B. CAV amounts in the samples were processed and evaluated using the software Empower 3 from Waters Corp. (Milford, MA, USA).

Empower 3 calculates the unknown sample concentrations (CAV_A_) by comparing the samples’ peak area responses with those provided by the standards, using an external calibration with a linear fit.

CAV recovery in the samples (C_R_) was calculated via weight correction from the Empower 3 data, including specific factors for the applied dilutions and considering the total expected CAV to be recovered. Initially, the theoretical sample’s CAV mass (CAV_M_) was calculated from the total sample weight (S_W_), considering the CAV loading in the sample (CAV_L_). This step is explained in Equation (2). In the following step, the theoretical sample’s CAV concentration was calculated by dividing CAV_M_ by the dilution factor (D_F_ in mL), and obtaining a concentration expressed in mg/mL. Afterward, the result was multiplied by 1000 to convert the result to µg/mL. Now, the result can be compared to CAV_A_ and the formula from Equation (3) can be employed:

(2) $$CAV_{M}=S_{W}\times CAV_{L}$$

(3) $$CAV_{R}=\frac{CAV_{A}}{\frac{CAV_{M}}{D_{F}}\times 1000}\times 100$$

Equations (2) and (3): CAV_A_ expressed in µg/mL, Sample weight and CAV_M_ expressed in mg. CAV_R_ is expressed in percentage. D_F_ is expressed in mL.

A calibration curve was previously proven in the range of 30 and 650 µg/mL in the context of a recently published and validated CAV method in the same analytical facility, utilizing the same UPLC Acquity H-Class system and column type [28]. Furthermore, this methodology was found to be accurate, precise and robust, in accordance with the current regulatory framework for analytical methods, including ICH Q2 (R2) [29] and Q14 guidelines [30].

Moreover, as complement data for this publication, analytical studies proved the current HME matrix’s specificity and accuracy. The specificity, or selectivity, was demonstrated by comparing the identifying chromatogram of CAV with those of the HME matrix and blank solution, confirming the absence of interfering peaks at the CAV’s retention time [31,32]. As the analyte is determined with a Photo-Diode Array detection, it was possible to establish whether additional species are co-eluting in the same retention time by analyzing the full spectral range of the detector during an injection, guaranteeing the peak purity. The chromatographic software addresses this by using the “Purity Flag” [32]. The accuracy in the presence of sample matrix, either with SOL or VA64 and preblended, by preparing solutions at three concentration levels at 0.5, 1.0 and 2.0% of CAV in triplicate, covering the reportable range. The response was evaluated regarding the mean percent recovery of CAV, and the acceptance interval was set between 98.0% and 102.0%.

To prove the UPLC system’s precision, system suitability testing was performed at the beginning and during every sequence, injecting a 200 µg/mL CAV solution six consecutive times, and the RSD (%) was successfully controlled below 2.0%.

Samples wise, approximately 15 mg (0.5% *w*/*w* total CAV) or 30 mg (1.0% *w*/*w* total CAV) and 2985 mg or 2970 mg of SOL or VA64 were accurately weighted in 100 mL of starting mobile phase (A:B = 90:10) and solubilized in an ultrasonic bath for 10 min, followed by magnetic stirring for 40 min and another 10 min in the ultrasonic bath. Prior to injecting into the UPLC, the samples were filtered through a nylon filter with a pore size of 0.45 µm, discarding the first 5 mL of solution.

CAV recovery was performed similarly to that described above, where 3000 mg of HME-SEDDS was accurately weighed, dissolved in 100 mL of the starting mobile phase (A:B = 90:10), and completely solubilized in an ultrasonic bath for 10 min. This was followed by magnetic stirring for 40 min and another 10 min in the ultrasonic bath. Before injecting into the UPLC, the samples were filtered through a nylon filter with a pore size of 0.45 µm, discarding the first 5 mL of solution.

## 4. Conclusions

In this work, two solid SEDDS preparation routes (introducing carvedilol in low doses of <1% *w*/*w* into the process in the solid or solubilized form) were explored in a pilot-scale twin-screw hot melt extruder. It was shown that for the purpose of achieving low-dose solid SEDDS with a uniform drug content in the final product, pre-dissolving CAV in the lipid SEDDS vehicle is superior to the conventional drug-matrix-preblend feeding.

A beneficial plasticizing effect of SEDDS was observed, signified by the reduction in torque with an increasing feeding rate of SEDDS and a reduction in T_g_ of the polymer matrix. DSC and WAXS analysis demonstrated a compatibility between the drug, the polymer and the SEDDS in the studied proportions, indicating that carvedilol remained in the dissolved or amorphous form. Emulsification properties of both Soluplus^®^ and Kollidon^®^ VA64 were adequate for potential oral delivery, with the mean droplet size of microemulsions being below or around 250 µm. The emulsification and reconstitution times were more rapid in the solid SEDDS with the Kollidon^®^ matrix, whereas the Soluplus^®^ samples showed sustained dissolution.

This work presents promising pioneer data while the full potential of solidification of SEDDS via HME for oral delivery of low-dosed poorly water-soluble drugs is yet to be further explored. Future studies will aim at this objective.

## Figures and Tables

**Table 1:** Basic characterization of blank and CAV loaded SEDDSs in demineralized water at 37 °C. Values ± SD except emulsification time and were automatically calculated with equipment software Kalliope^®^ version 2.22.2 (Anton Paar, Graz, Austria).

Parameter	Blank SEDDS		CAR-SEDDS (5% *w*/*w*)	
Concentration [*w*/*v*]	0.4%	2.0%	0.4%	2.0%
Droplet size [nm]	35.7 * ± 3.1	36.9 * ± 4.2	44.7 * ± 5.4	49.0 * ± 4.8
Polydispersity index	0.048 * ± 0.03	0.052 * ± 0.04	0.088 * ± 0.02	0.101 * ± 0.05
Zeta potential [mV]	−3.48 ± 0.98	2.59 ± 0.92	1.12 ± 0.96	0.92 ± 0.90
Transmittance [%]	73.6 ± 4.2	74.5 ± 2.8	77.3 ± 3.5	63.5 * ± 3.3
Emulsification time [s]	12 ± 3	15 ± 2	16 ± 3	15 ± 4
Appearance	Bluish clear	Bluish clear	Bluish clear	Bluish clear

* *p* < 0.05.

**Table 2:** Particle size distribution (PSD) of raw materials. The values are mean ± SD (n = 3).

Sample	D_10_	D_50_	D_90_	Span
Carvedilol	2.9 ± 0.03	11.3 * ± 0.1	27.6 ± 0.3	2.2
Kollidon^®^ VA64	38.8 ± 0.6	98.9 * ± 1.5	171.9 ± 2.8	1.3
Soluplus^®^	216.8 ± 12.6	337.9 * ± 12.4	481.9 ± 10.0	0.8

* *p* < 0.05.

**Table 3:** CAV recovery in preblends and in HME-SEDDS. (N/A = not applicable).

Sample	CAV in Preblend [%]	CAV in HME-SEDDS [%]
1_SOL-10%_PREBLEND_0.5%_CAV	96.75 ± 2.61	79.35 ± 0.24
2_SOL-20%_PREBLEND_1.0%_CAV	37.04 ± 0.50	53.29 ± 0.11
3_VA64-10%_PREBLEND_0.5%_CAV	76.44 ± 0.63	66.16 ± 0.15
4_VA64-20%_PREBLEND_1.0%_CAV	81.04 ± 0.79	110.07 ± 0.48
5_SOL-10%_SEDDS_0.5%_CAV	N/A	76.08 ± 10.47
6_SOL-20%_SEDDS_1.0%_CAV	N/A	96.98 ± 0.70
7_VA64-10%_SEDDS_0.5%_CAV	N/A	98.18 ± 1.85
8_VA64-20%_SEDDS_1.0%_CAV	N/A	97.21 ± 8.83

**Table 4:** Raw materials used for preparation of SEDDS and final HME-SEDDS with respective glass transition temperatures (T_g_), melting points (T_m_) and degradation temperatures (T_d_). The amounts of each material in SEDDS and HME-SEDDS are provided in the given ranges, corresponding to 10–20% *w*/*w* SEDDS and 80–90% *w*/*w* polymers in the final formulation.

Raw Material	Description	T_g_/T_m_ [°C]	T_d_ [°C]	SEDDS [% *w*/*w*]	HME-SEDDS [% *w*/*w*]
Carvedilol	poorly water-soluble BCS class II drug substance	115 [13]	248 [25]	5	0.5–1.0
Labrafac^®^ lipophile	Medium-chain Triglycerides Liquid lipid for CAV solubilization	<−5 *	>250 *	30	3–6
Kolliphor^®^ RH40	PEG-40 hydrogenated castor oil Surfactant for CAV solubilization	16–26 *	300 *	30	3–6
Capmul^®^ MCM	Glyceryl caprylate/caprate Type I Liquid lipid for CAV solubilization	25 [5]	148.9 *	30	3–6
Transcutol^®^	Diethylene Glycol Monoethyl Ether Co-solvent for CAV solubilization	−80 *	196–200 *	10	1–2
Kollidon^®^ VA 64	Vinyl pyrrolidone: mvinyl acetate 6:4 Polymer matrix for liquids SEDDS adsorbtion	105 [26]	270 [26]	N/A	80–90
Soluplus^®^	Poly(vinylcaprolactam-covinylacetate ethylene glycol)graft polymer Polymer matrix for liquids SEDDS adsorbtion	72 °C [26]	278 °C [26]	N/A	80–90

* melting, boiling and degradation temperatures of liquid raw materials were found in manufacturers’ safety data sheets.

**Table 5:** Formulations prepared in the present study via continuous HME in the split feeding setup.

CAV Introduction	Sample	Polymer [%]	SEDDS * [%]	CAV Total [%]
PREBLEND	1_SOL-10%_PREBLEND_0.5%_CAV	90% Soluplus^®^	10	0.5
	2_SOL-20%_PREBLEND_1.0%_CAV	80% Soluplus^®^	20	1.0
	3_VA64-10%_PREBLEND_0.5%_CAV	90% Kollidon^®^ VA-64^®^	10	0.5
	4_VA64-20%_PREBLEND_1.0%_CAV	80% Kollidon^®^ VA-64^®^	20	1.0
SEDDS	5_SOL-10%_SEDDS_0.5%_CAV	90% Soluplus^®^	10	0.5
	6_SOL-20%_SEDDS_1.0%_CAV	80% Soluplus^®^	20	1.0
	7_VA64-10%_SEDDS_0.5%_CAV	90% Kollidon^®^ VA-64^®^	10	0.5
	8_VA64-20%_SEDDS_1.0%_CAV	80% Kollidon^®^ VA-64^®^	20	1.0

* SEDDS composition (*w*/*w*): 5% CAV, 28.5% Labrafac^®^ lipophile, 28.5% Kolliphor^®^ RH40, 28.5% Capmul^®^ MCM, 9.5% Transcutol^®^.

## Data Availability

The original contributions presented in the study are included in the article, further inquiries can be directed to the corresponding authors.

## Abbreviations

ASD	Amorphous solid dispersion
CAV	Carvedilol
DSC	Differential scanning calorimetry
HME	Hot melt extrusion
HME-SEDDS	Solid SEDDS prepared by hot melt extrusion
HPC	Hydroxy propyl cellulose
HPMC	Hydroxypropyl methylcellulose
HPMCAS	Hydroxy propyl methylcellulose-acetate/succinate
LBDS	Lipid-based delivery systems
MCC	Microcrystalline cellulose
PDI	Polydispersity index
PSD	Particle size distribution
RSD	Relative standard deviation
SEDDS	Self-emulsifying drug delivery systems
SMEC	Specific mechanical energy consumption
SOL	Soluplus^®^
SOL-HME-SEDDS	Solid SEDDS prepared by hot melt extrusion with Soluplus^®^
UPLC	Ultra-High-performance liquid chromatography
VA-64	Kollidon^®^ VA-64, copovidone
VA-64-HME-SEDDS	Solid SEDDS prepared by hot melt extrusion with Kollidon^®^ VA-64
WAXS	Wide-angle X-ray scattering
